# Supplementary material for: Glutamatergic basolateral amygdala to anterior insular cortex circuitry maintains rewarding contextual memory
Source: Commun Biol. 2020 Mar 20;3:139. doi: 10.1038/s42003-020-0862-z (PMC7083952; doi:10.1038/s42003-020-0862-z)
Supplement: Supplementary file 1 — Supplementary Information [file 42003_2020_862_MOESM1_ESM.pdf]

SUPPLEMENTARY FIGURES

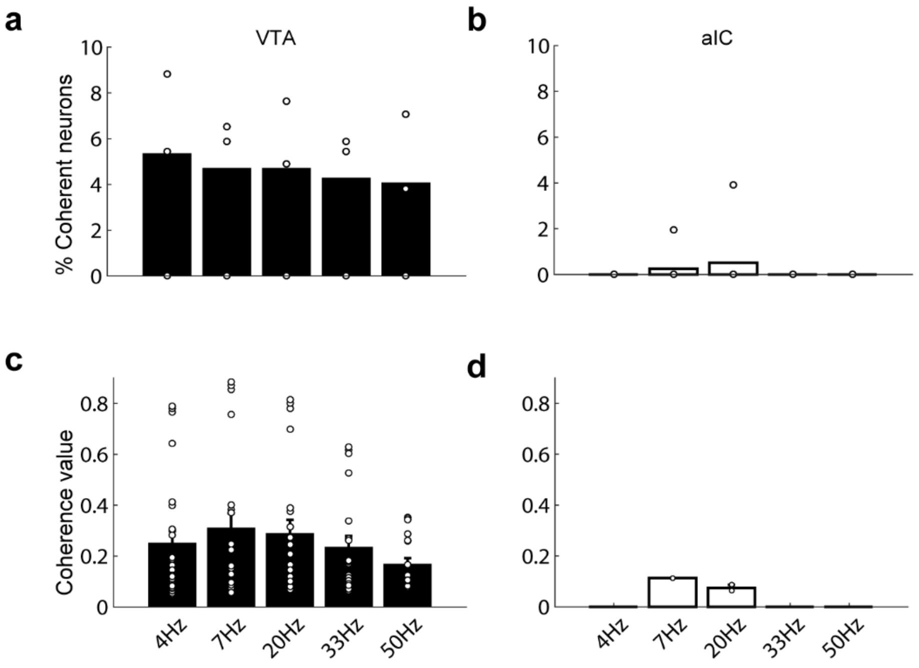

Supplementary Figure 1. Percentage of coherent neurons during optogenetic stimulation at different laser frequencies. a) Percentage of neurons with spike-laser coherence recorded in VTA. Each dot represents values from a single mouse. And in panel **b)** coherence from neurons recorded in aIC. **c)** and **d)** the average coherence value of all neurons with a coherence recorded in VTA and aIC, respectively (a coherence value of 1 indicates a perfect correlation between spikes activity and laser frequency, whereas a 0 shows no correlation. Note that the spike-laser coherence analysis confirmed that VTA neurons, but not aIC neurons, could fire phase-locked at up to 20 Hz laser stimulation, but at higher frequencies, coherence values gradually dropped or were absent in aIC. In c) and d) each dot represents an individual neuron.

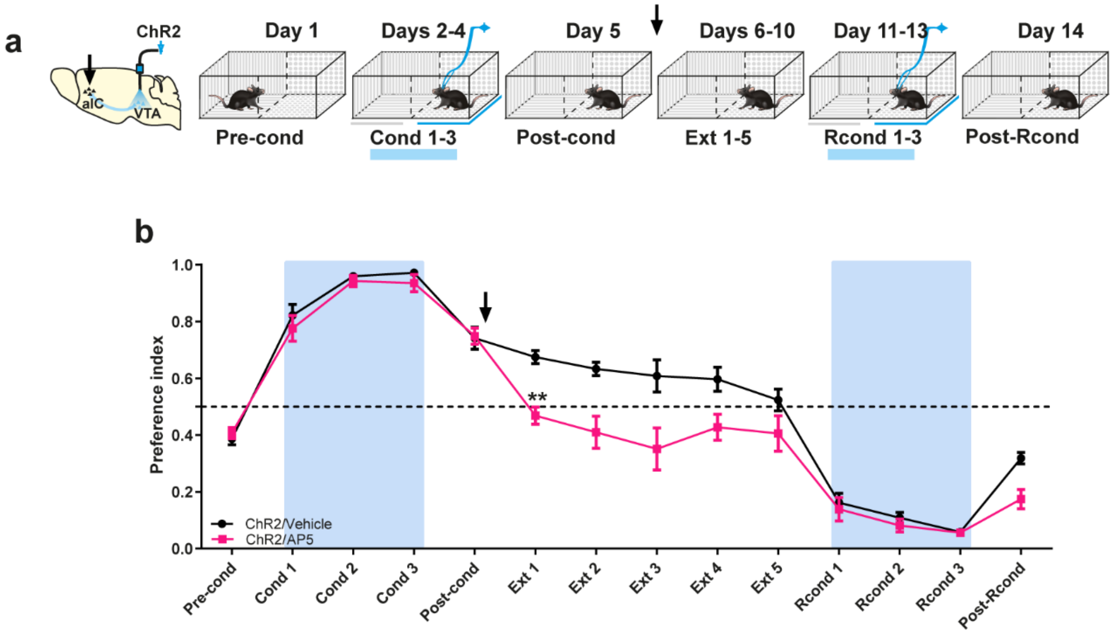

Supplementary Figure 2. Blockade of NMDA receptors in the aIC does not impair formation of new conditioning. a) Left, diagram of virus injection and photoactivation into VTA TH+ neurons. Right, timeline of behavioral procedures. The preference of the compartments was determined during the pre-conditioning (Pre-cond). Mice were conditioned during 3 sessions 20 min of photoactivation of VTA TH+ neurons (Cond 1-3). Blue bars show photoactivation of VTA with the blue laser. In the post-conditioning (Post-cond), preference of the compartments was tested after optogenetic stimulation conditioning. **b)** After place preference was extinguished (Ext 1-5), the mice were conditioned with photoactivation of VTA to the opposite side (Rcond 1-3). Two-way repeated measures ANOVA with Greenhouse-Geisser Correction Factor showed main group effect: $F_{1,11}$=20.17, *P*=0.0009, time effect: $F_{4.124,45.37}$=133.2, *P*<0.001, and interaction: $F_{13,143}$=3.467, *P*=0.0001. Post- hoc Bonferroni test ChR2/Vehicle (n=6) vs ChR2/AP5 (n=7) for Ext 1 *P*=0.0034. All data are shown as mean ± SEM. The arrow indicates the time of administration of the drugs. Dashed horizontal line indicates no preference. ***P*<0.001.

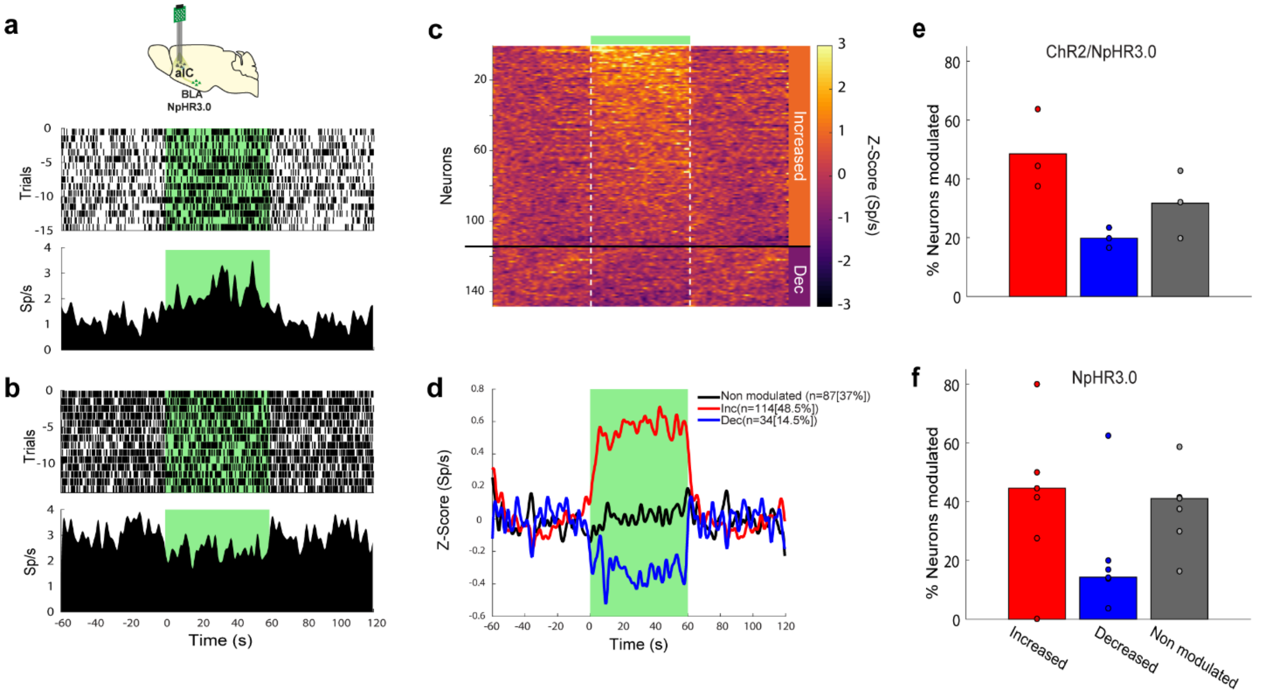

Supplementary Figure S3. Expression NpHR in BLA evoked disinhibition of local cortical circuits in the aIC. **a, b)** Sample raster plots and Z-Score normalized color-coded PSTH of all excited (a) and inhibited (b) neurons recorded in the aIC while the glutamatergic BLA-aIC fibers were opto-inhibited with NpHR3.0 in BLA. **c)** Yellow colors indicate higher firing rate whereas dark colors inhibitory responses. White vertical dashed lines depict the start and the end of photoinhibition of green laser, which was constant pulsed at 12-14 mW and 532 nm. **d)** Population average of Z-scores for neurons categorized by response type (increased, decreased, non-modulated) during photoinhibition. **e)** Comparison of percentage of neurons modulated during the photoinhibition in the double injected mice (ChR2/NpHR3.0) and in **f)** single injected mice (NpHR3.0) in BLA. Each dot is a single mouse.
